# New trends in developmental coordination disorder: Multivariate, multidimensional and multimodal

**DOI:** 10.3389/fpsyt.2023.1116369

**Published:** 2023-01-27

**Authors:** Li Ke, Xueting Su, Sijia Yang, Zhihao Du, Shunsen Huang, Yun Wang

**Affiliations:** ^1^Collaborative Innovation Center of Assessment for Basic Education Quality, Beijing Normal University, Beijing, China; ^2^Chinese PLA Center for Disease Control and Prevention, Beijing, China; ^3^State Key Laboratory of Cognitive Neuroscience and Learning, Beijing Normal University, Beijing, China; ^4^College of Physical Education and Sports, Beijing Normal University, Beijing, China

**Keywords:** developmental coordination disorders, bibliometrics, visual analysis, cluster analysis, neuroimaging

## Abstract

Developmental coordination disorder (DCD) is a motor development disorder that affects an individual’s growth and development, and may persist throughout life. It is not caused by intellectual or physical disability. Studies have suggested DCD often occurs in childhood, resulting in a series of abnormal manifestations that hinder children’s normal development; cohort studies suggest a higher incidence in boys than in girls. Early diagnosis and appropriate interventions can help relieve symptoms. Unfortunately, the relevant research still needs to be further developed. In this paper, we first start from the definition of DCD, systematically investigate the relevant research papers in the past decades and summarize the current research hotspots and research trends in this field. After summarizing, it is found that this research field has attracted more researchers to join, the number of papers published has increased year by year and has become a hot spot in multidisciplinary research, such as education, psychology, sports rehabilitation, neurobiology, and neuroimaging. The continuous development of the correlation between perinatal factors and DCD, various omics studies, and neuroimaging methods also brings new perspectives and working targets to DCD research. DCD-related research will continue to deepen along the research direction of multivariate, multidimensional, and multimodal.

## Introduction

Motor development is essential for children’s physical and mental development. Developmental coordination disorder (DCD) is a motor development disorder characterized by inadequate motor performance, which affects individuals’ activities of daily living and academic performance, and cannot be explained by intellectual disability or any congenital/acquired neurological condition. DCD occurs more commonly in children, with detection rates generally ranging from 5% to 20%, and epidemiological findings indicate that boys are usually more likely to be detected than girls, with male to female ratios ranging from 2:1 to 7:1 (1–4). As a limiting condition, DCD seriously affects children’s daily lives (5). These difficulties not only cause problems in children’s motor development, increase the risk of attention, social skills, reading, and spelling difficulties, but also affect children’s self-esteem and motivation development, and they are easily bullied (6), resulting in a series of abnormal manifestations that hinder children’s normal development.

For some children, difficulty in movement is a single symptom; for others, deficits in motor coordination are just one of many problems, and may also involve deficits in speech and language, reading, attention, and/or social and emotional aspects (7–10). The formation of DCD in children may be affected by various factors, such as self-status, maternal pregnancy and childbirth, family, and school environment, etc. (11–13); Although the role of neural factors in the formation of DCD has been increasingly noted with the progress of cognitive neuroscience, the mechanism is still unclear. But it is certain that motor development disorders do not disappear as children age (14, 15); without intervention, these problems can extend from childhood into adulthood and, more seriously, affect an individual’s growth and development. Various interventions, especially those that work at the cognitive level of children, can have a certain effect on children’s motor development (16). Therefore, early detection and intervention of DCD can support the development of children and adolescents as early as possible and avoid long-term adverse effects.

## Unity of definition

The field of motor development research initially focused on the difficulties and deficits to movement in children, often without a uniform name. The earliest researchers often used the term “clumsy” to describe children with difficulty with movement or motor acquisition (17, 18).

Subsequently, researchers have called such children “dyspraxia,” “motor learning difficulties,” and “Disorder of Attention, Motor Control, and Perception” (DAMP) from different perspectives, such as neurology. However, the above terms either focus on a specific aspect or only focus on the child’s symptoms and do not refer to the child’s development process (19). Some medical diagnostic criteria, international conferences, and documents have gradually tried to define terminology for them, such as “DCD,” “specific motor skill development disorder,” etc. Until 2013, the latest edition of the Diagnostic and Statistical Manual of Mental Disorders (5th ed.) (DSM-V) included DCD as a neurodevelopmental disorder, cataloging motor disorders under this category and giving four detailed diagnostic criteria:

(A) The acquisition and execution of coordinated motor skills is substantially below that expected given the individual’s chronological age and opportunity for skill learning and use. Difficulties are manifested as clumsiness (e.g., dropping or bumping into objects) as well as slowness and inaccuracy of performance of motor skills (e.g., catching an object, using scissors or cutlery, handwriting, riding a bike, or participating in sports).

(B) The motor skills deficit in Criterion A significantly and persistently interferes with activities of daily living appropriate to chronological age (e.g., self-care and self-maintenance) and impacts academic/school productivity, prevocational and vocational activities, leisure, and play.

(C) Onset of symptoms is in the early developmental period.

(D) The motor skills deficits are not better explained by intellectual disability (intellectual developmental disorder) or visual impairment and are not attributable to a neurological condition affecting movement (e.g., cerebral palsy, muscular dystrophy, and degenerative disorder) (1).

## Increasing interest in developing countries

**FIGURE 1:**
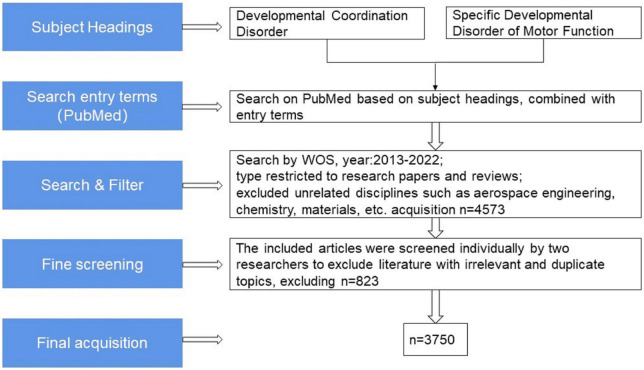
Data acquisition and entry process.

**FIGURE 2:**
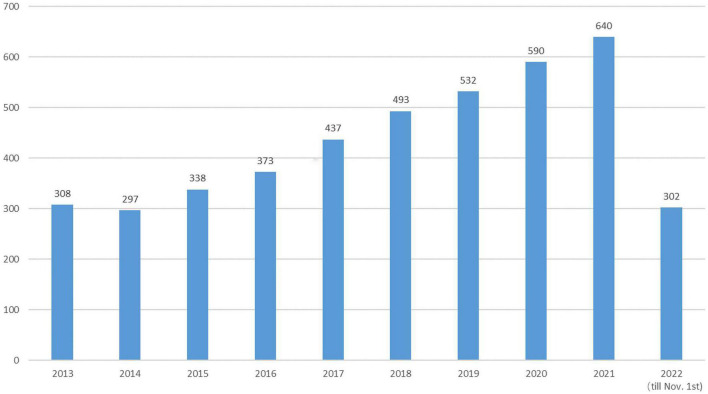
Annual number of articles related to children’s developmental coordination disorder (DCD).

Since then, the field of research on DCD has steadily developed. We grabbed the English literature related to the field of children’s DCD research from January 2013 to November 2022 in the Web of Science (WOS) Core Collection database for bibliometric analysis (20) and removed the Web of Science category and a small number of journals in the fields of aerospace engineering, chemistry, materials, economics, computer and other obvious topics, with a total of 3750 articles meeting the target conditions (see Figure 1). The analysis shows that the annual number of children’s DCD research papers published decreased slightly compared with 2013 in 2014, and the overall trend from 2013 to 2021 showed an upward trend year by year, and the number of papers published in 2021 (640) has reached twice the number published in 2013 (308), and as of 1 November 2022, the number of papers published in 2022 has reached 302 (see Figure 2).

**FIGURE 3:**
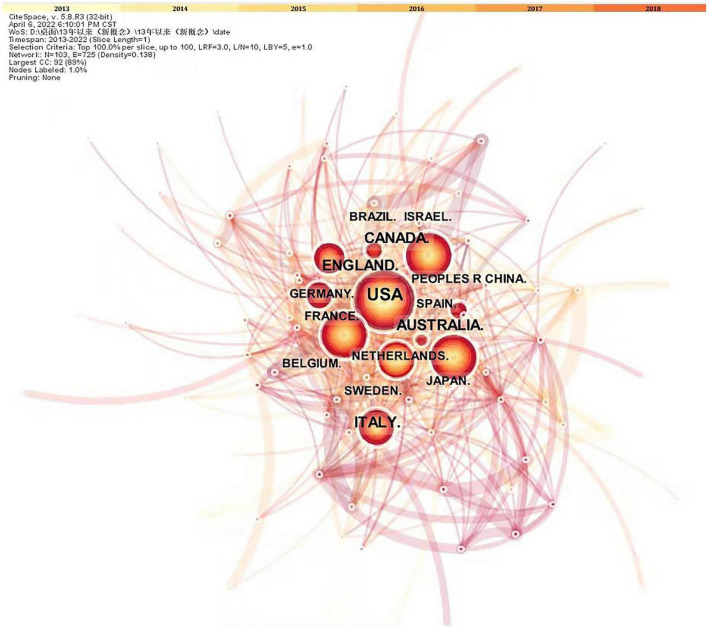
Research papers published on state relations. The nodes in the graph are countries; the larger the node, the higher the volume of articles issued; and the connecting lines indicate the cooperation between countries; the more connecting lines, the better the communication ability of the nodes.

Research on neurodevelopmental disorders, including DCD, is critical to protecting children’s wellbeing and improving the quality of the population. Analysis of the countries or regions where papers were published shows that the United States ranks first in the field of children’s DCD research in terms of the number of papers published; the number of papers published far exceeds that of other countries, and the research contribution is in a central position. Eight of the top 10 countries in terms of number of publications and centrality are developed countries, indicating that the current research on DCD in children is mainly dominated by developed countries (see Figure 3 and Tables 1, 2). The strong economic base of developed countries underpins their focus on child development. In recent years, China and Brazil have gradually emerged in the field of DCD, and China has ranked 6th in the number of articles published so far, about 230. Brazil ranked 10th with 151 publications. China and Brazil also rank in the top 10 research centers, indicating that developing countries are also deepening their understanding of childhood DCD, and their status and role in this research field are becoming more prominent. As populous countries and rapidly emerging economic entities, developing countries are set to play an increasingly important role in the field of neurodevelopmental research in children, including DCD research.

**TABLE 1:** The top 10 countries/regions in the country centrality of children’s developmental coordination disorder (DCD) research field.

Ranking	Centrality	Country/Regions
1	0.26	United States
2	0.15	England
3	0.1	Australia
4	0.1	Italy
5	0.09	Germany
6	0.08	Canada
7	0.08	Netherlands
8	0.07	Peoples R. China
9	0.05	France
10	0.04	Brazil

**TABLE 2:** The top 10 countries/regions in the number of children’s DCD research papers published.

Ranking	Number of publications	Country/Regions
1	1215	United States
2	403	England
3	394	Canada
4	387	Australia
5	316	Italy
6	230	Peoples R. China
7	219	Netherlands
8	217	Germany
9	208	France
10	151	Brazil

## Perspectives from different research fields

**FIGURE 4:**
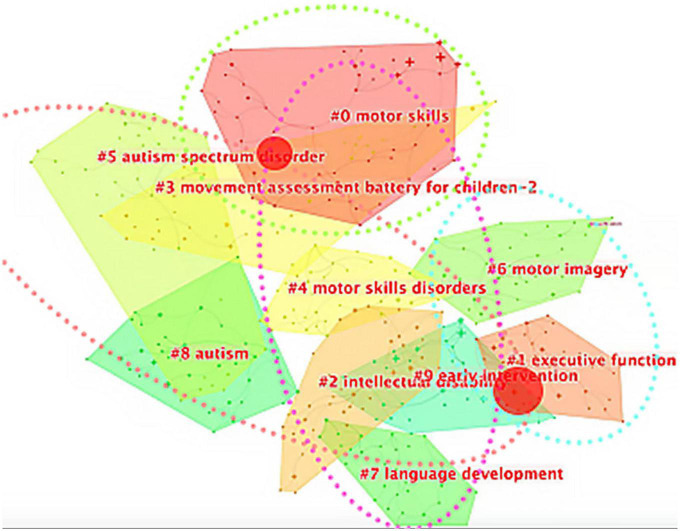
Keywords cluster analysis. The blue circle is plate 1: # 1 and # 6 make up the cognitive neuroscience plate (relationship between cognitive function and process and explicit DCD); the red circle is plate 2: # 5, # 3, # 4, # 8, # 2, and # 9 constitute the medical plate (co-occurrence evaluation intervention of NDDs); the pink circle is plate 4: # 0, # 4, # 2, and # 7 constitute the psychology plate; the green circle is plate 3: # 0 is an education plate.

Compared to language development and so on, children’s motor development occurs earlier in early childhood and is easier to observe, meaning there are some intrinsic correlations between motor acquisition and various learning mechanisms in school age. Therefore, research in this field attracts researchers from different disciplinary backgrounds. We conducted keyword cluster analysis on the relevant English literature in the field of childhood DCD research from 2013 to the present and formed a total of 10 clusters: #0 motor skills, #1 executive function, #2 intellectual disability, and #3 movement assessment battery for children-2, #4 motor skill disorders, #5 autism spectrum disorder, #6 motor imagery, #7 language development, #8 autism, #9 early intervention (see Figure 4). The above clusters can be clustered and further divided into four plates: #1/#6 focusing on the cognitive neuroscience underpinnings of children’s motor development; #2/#3/#4/#5/#8/#9 focusing on the assessment, diagnosis, and intervention of specific disorders, as well as comorbid conditions for DCD and other NDDs such as autism; #2/#7 exploring the relationship between children’s motor development and other areas of child development, such as language; #0 focusing more on teaching strategies and learning mastery of motor skills.

Among them, the research on the cognitive neural basis of children’s motor development mostly comes from the research perspective of cognitive neuroscience. The evaluation and diagnosis interventions for DCD mostly come from the research perspective of pediatrics, rehabilitation, and other clinical medical disciplines. The research on the developmental status and developmental influencing factors of children with DCD mostly comes from the research perspectives of developmental psychology, experimental psychology, multidisciplinary psychology, and other psychological disciplines. The research on physical activity intervention and physical education psychology mostly comes from the research perspective of special education, exercise science, and other pedagogical disciplines.

The above analysis results reflect the current differences in the focus of different disciplines on the research of children’s motor development disorders. Specifically, physical education pays more attention to the decomposition and training mastery of motor skills, such as Smith et al. (21), who reviewed the differences in gait between children with and without DCD when walking and running; a study conducted in Pakistan showed that trampoline exercise training was associated with improved exercise performance in children with DCD (22). Psychology researchers, on the other hand, focus more on motor development milestones, influencing factors, and developmental outcomes, such as Bolk et al. (23), who revealed that extremely preterm birth is a high-risk factor for DCD and related comorbidities in children; Karras et al. (24), who focused on and studied health-related quality of life in children with DCD.

The discipline of clinical medicine pays more attention to the assessment, diagnosis, and intervention of specific disorders, such as in the journal Archives of Disease in Childhood, Kirby et al. (25) discussed the diagnosis of DCD, including a comprehensive understanding of past history, clinical observation, and the use of standardized assessment tools; In a journal in the pediatric category, research on virtual reality tools for interventions in children with DCD was reviewed (26).

The discipline of cognitive neuroscience pays more attention to the neural mechanisms and patterns of motor development, such as that studied by Rinat et al. (27) using resting-state functional MRI to explore the similarities and differences in brain functional connections between DCD children and normally developing children; Studies have found that children with DCD generally have deficits in language synchronization to auditory and visual routine stimuli compared to normally developing children, and that the stability of audio-verbal synchronization during practice is associated with the thickness of the sensorimotor cortex (28).

In addition, between the above four sections, there are two nodes that reflect the cross-convergence of different research angles in the research focus, namely “Children’s Motor Development Assessment Tool Technology” and “Early Intervention of Children’s Motor Development,” which are the focus of interdisciplinary common concern. These two aspects are also the places where there is much debate between disciplines. For example, there is widespread controversy about the diagnostic criteria and evaluation tools for DCD, one of which is that it is difficult for researchers to determine whether the NDDs are a single barrier or a subtype in terms of detection criteria. There are common comorbidities among all types of NDDs, which some researchers believe may indicate many types (8), but others conversely believe that since difficulties/disorders of motor in children are a universal feature in various types of NDDs and even neurodevelopmental disorders like cerebral palsy (29), it may not even be an independent disorder (30).

Secondly, there is currently no tool that can be used as the “gold standard,” and only the widely used tools with standardized norms should be selected for evaluation. The jury is still out on the advantages and disadvantages of standardized assessment tools and scales. Standardized assessment tools may be interfered with by other factors in assessing athletic performance (31). There is a lack of operability in the diagnostic criteria of “significant interference with academic achievement or activities of daily living” (4, 6), while this type of information is difficult to obtain in standardized assessment tools but can be obtained from scale-based tests (32). However, scale-type tests are currently generally used as an adjunct to detection in clinical samples and are recommended as a screening tool in general population at best, even though the most well-documented scale-type tests such as DCDQ are still less sensitive (31, 32).

The intervention of DCD, from the theoretical basis and method of intervention, is generally classified into three paths: process-oriented, task-oriented, and traditional physical and occupational therapy (33). Interventions in the process-oriented approach focus on the components of the movement itself and the child’s bodily functions. It aims to improve children’s skill performance by paying attention to the details of movement breakdown (34). Sensory integration training is in this category. However, current evidence suggests that process-oriented training is not significant (35, 36). The task-oriented approach is to improve children’s motor performance based on specific tasks that cause motor difficulty in children, considering the fundamentals of motor control/motor learning and ecology (35). Cognitive Orientation to Daily Occupational Performance (CO-OP) falls into this category, and this intervention program is significantly more effective than other interventions (37). The traditional physical and functional therapy approach relies on the basic assumption that motor skills have developmental ladder. The intervention focuses on basic training of gross motor/fine motor, and the development of these basic motor abilities as a prerequisite for motor skill development tends to incorporate some task-oriented approaches (35). But what the active ingredients of its intervention are also controversial. Some researchers believe that the active ingredients of the intervention may be cognition (38–40), sociality (39, 41), or experience/practice (42, 43), and the nature and guidance of activities (13, 43, 44). Among them, the most discussed is the importance of cognition to the intervention effect in children with DCDs. By comparing different intervention methods, Sims et al. (45) believe that the most effective part of the intervention is the process involving cognitive function, that is, the process that facilitates the acquisition, processing, combination, planning, and construction of information in children; In recent years, new cognitive methods of motor imaging training have been arguing that appropriate exercise programs are important for safe and efficient performance of activities of daily living (40). Sugden and Chambers (33) point out that the process-oriented approach and the task-oriented approach have different theoretical starting points but employ some very similar interventions. These measures often point to mental processes such as perception, memory, attention, and planning. Cognitive-directed occupational therapy is generally the acquisition of skills using cognitive strategies (46). With the deepening of the discussion of the mechanism of DCDs, researchers gradually began to map specific intervention tasks to potential cognitive factors and discuss the effectiveness of interventions.

## Current hotspots and research trends

**FIGURE 5:**
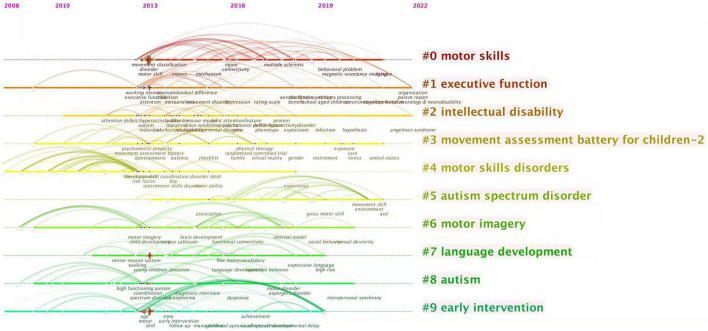
Co-current zone of keywords analysis. The nodes on the ten horizontal lines in the diagram are the keywords and their corresponding occurrence times, the rightmost labels are the keyword clusters, and the keyword clusters from #0 to #9 are arranged in descending order of the number of keywords.

**FIGURE 6:**
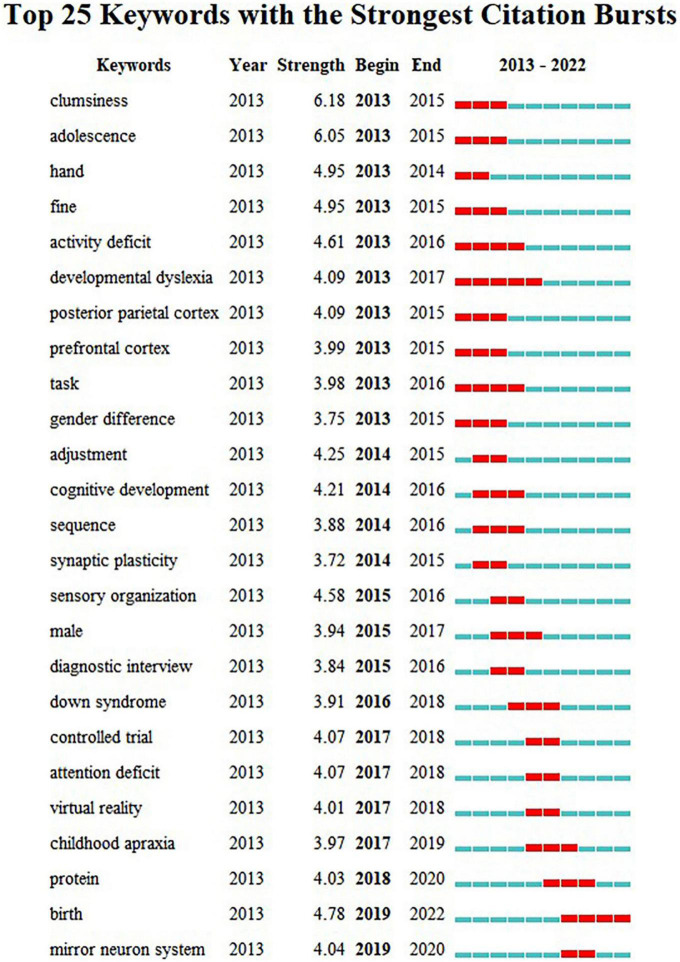
Burst detection of keyword analysis. Each year from 2013 to 2022 is set as a time zone division, with the blue line in the diagram indicating the time interval and the red line indicating the time period when the keyword burst is detected.

In recent years, the research topics related to DCD have also been constantly changing. In general, keywords show the frontiers of research on this topic (47), and we analyze the above collections by year (see Figures 5, 6).

According to the analysis of keyword co-current zone, the research hotspots of clinical medicine perspective in recent years are “Comorbid symptoms” and “Behavior disorder.” The research hotspots of psychology perspective are “Parental involvement,” “Intervention in the environment,” “Perinatal intervention,” and “DCD children’s social expression behavior.” The research hotspots of cognitive neuroscience perspective are “Nuclear magnetic resonance (NMR),” “Brain image,” and “Neural mechanism study.” The research hotspots of physical education perspective are “The study of motor skills” and “Improvement of gross motor skills.” New keywords including “neurocognitive,” “proteomics,” “perinatal” have appeared frequently in the past three years. Further according to the analysis of keyword burst detection, the top 25 keywords with the strongest sudden outbreak obviously show a certain time stage. In the past 5 years, the emerging keywords of new outbreaks are: “mirror neuron system,” “protein,” “birth.” The above results reflect the current interest in neurodevelopmental mechanisms, perinatal factors and proteomics in the field of pediatric DCD and reflect the increasing tendency of research to start from behavioral-brain-cellular molecular data when understanding the development process of children.

To study the neurodevelopmental mechanism of DCD, some studies have used brain imaging technology to explore and find that DCD has changes in brain structure and function. A study on brain structure used graph theoretical analysis to reveal differences in global and regional topological attributes of the brain structure network, which showed that children who met the criteria for DCD and ASD showed more widespread topological changes than those with DCD symptoms alone (48).

On resting functional MRI, Rinat et al. (27) found that children with DCD showed altered functional connectivity between the sensorimotor network and the posterior cingulate cortex (PCC), precuneus, and posterior middle temporal gyrus (pMTG). Fuelscher et al. (49) used activation likelihood estimation (ALE) to perform a meta-analysis of seven studies using fMRI and found that, compared to the control group, children with DCD had reduced in activation in middle frontal gyrus, superior frontal gyrus, cerebellum, supramarginal gyrus, and inferior parietal lobule when performing manual dexterity tasks.

On diffusion tensor imaging (DTI), Langevin et al. (50) found white matter parameters changed in children with ADHD and DCD. Further researchers found that neurodevelopmental disorders such as DCD may be related to white matter development delay and/or structural disorders, and the DCD children are more prone to abnormal white matter development than ordinary children (51–53), correspondingly with relatively higher detection rates of various types of NDDs. Moreover, there are differences in the pattern of white matter abnormalities in children with different NDDs such as DCD and ADHD. In studies of preterm very low birth weight (VLBW) children, abnormalities in the white matter structure of children with DCD were mostly present in white matter in areas related to motor function, including corticospinal tracts, cerebellar tracts, and cerebellum (51). White matter retardation in ADHD is mainly manifested in the frontostriatal and superior longitudinal fasciculus (54).

Other studies have shown that perinatal factors such as preterm birth and VLBW greatly increase the risk of children developing various neurological injuries, such as cerebral palsy, and also increase the risk of NDDs in K-12 children. Powls et al. (55) reported that VLBW children had more significant motor impairments at 6 and 8 years of age than controls; a review by Williams et al. (56) showed that the combined estimate of mild to moderate motor impairment in preterm infants was 40.5/100, and the composite estimate for moderate sports impairment was 19.0/100. An Italian study showed that preterm children scored significantly lower on DCDQ-Italian than control children, and 30% of them were at risk for DCD (57). In a study exploring brain structure and neurobehavioral function in preterm infants, it was discussed that changes in white matter parameters may also be associated with white matter development and myelination (58). Whether this is related to protein expression requires further research.

Studies are beginning to try to combine perinatal and brain imaging. For example, Grunewaldt et al. (59) reported that early ELBW children without CP with abnormal motor skills had significantly smaller white matter volume and cortical surface-area at age 10; Coker-Bolt et al. (60) conducted exercise assessment (at term and at 12 weeks corrected age) and DTI and DKI (mean corrected gestational age 42.2 ± 1.5 weeks) and there were significant differences in FA values in many regions of the left hemisphere between infants with poor and average performance on exercise assessment; while Kelly et al. (58) measured brain structure and neurobehavioral function in preterm infants at term-equivalent age (TEA), showing that there was no overall difference in brain structure-function relationship between very preterm and moderate-late preterm infants; Gui et al. (61) did two MRI scans of preterm infants at birth and TEA, providing information on brain development from birth to TEA, and models showing brain tissue capacity at birth and TEA helped predict motor outcomes at 18−24 months. We briefly summarize the factors and results of the above studies considering brain imaging and perinatal factors in the Table 3.

**TABLE 3:** Summary of five studies combining motor, perinatal, and brain imaging.

Title of paper	Age of participants	Grouping of participants	Number of participants	Behavioral tests	Brain imaging	Main results
Very preterm children at risk for developmental coordination disorder have brain alterations in motor areas (51)	TEA and 7 years	Very preterm or low birthweight children at risk of DCD (MABC2 ≤ 16 percentile) and children without risk of DCD	The number available for MRI at TEA was 160 (52 children at DCD and 108 children at non-DCD); The number available for MRI at age 7 was 125 (35 children at DCD and 90 children at non-DCD); The number available for DTI at age 7 was 137 (39 children at DCD and 98 children at non-DCD).	MABC-2; Wechsler Abbreviated Scale of Intelligence; Briggs-Nebes modified version of Annett’s Handedness Inventory.	MRI acquired at TEA; MRI and DTI acquired at age 7.	At term equivalent age, smaller brain volumes were found for total brain tissue, cortical gray matter, cerebellum, caudate accumbens, pallidum and thalamus in children at risk for developmental coordination disorder (*p* < 0.05); similar patterns were present at 7 years. There was no evidence for catch-up brain growth in at-risk children. At 7 years, at-risk children displayed altered microstructural organization in many white matter tracts (*p* < 0.05).
Follow-up at age 10 years in ELBW children−Functional outcome, brain morphology and results from motor assessments in infancy (59)	3 months corrected age and 10 years	Extremely low-birth-weight (ELBW) children and term-born children	At 3 months of corrected age, 31 ELBW children and 33 term-born children; At age 10, 23 ELBW children without cerebral palsy and 33 term-born children; At age 10, the number of available for MRI of ELBW children without cerebral palsy was 21, term-born children was 30.	At 3 months of corrected age: Prechtl’s General-Movement-Assessment; At age 10: Wechsler Intelligence Scale for Children, version-III (WISC-III); Stroop color word; Tower of London test; Trail-Making test; Beery–Buktenica Developmental Test of Visual–Motor Integration (Beery-VMI); MABC-2; ADHD Rating Scale—IV; Behavioral Rating Inventory of Executive Function (BRIEF)	MRI acquired at age 10.	The non-CP ELBW children had similar full-IQ but poorer working memory, poorer motor skills, and more attentional and behavioral problems compared to controls. On cerebral MRI reduced volumes of globus pallidus, cerebellar white matter and posterior corpus callosum were found. Cortical surface-area was reduced in temporal, parietal and anterior-medial-frontal areas. Poorer test-results and reduced brain volumes were mainly found in ELBW children with fidgety movements combined with abnormal motor-repertoire in infancy.
Correlating early motor skills to white matter abnormalities in preterm infants using diffusion tensor imaging (60)	At term and at 12 weeks and at 42 ± 1.5 weeks corrected age.	Infants classified as average (low-risk) and below average (high-risk) according to TIMP score at 12 weeks corrected age	Twenty-six preterm infants (high-risk infants 13, low-risk infants 13)	At term and 12 weeks CA using the Test of Infant Motor Performance (TIMP).	DTI or DKI acquired at 42 ± 1.5 weeks corrected age.	Significant differences were found between infants with poor vs. average performance on motor assessments at 12-weeks and FA values in several left hemispheric WM tracts (*p* < 0.05). High FA of the left anterior limb of the internal capsule (ALIC) predicted mean increase in TIMP scores on specific items for head lift in prone and head lift turn to sound (*p* = 0.045 and *p* = 0.002).
Brain structure and neurological and behavioral functioning in infants born preterm (58)	TEA	Infants born very (24–29 weeks) and moderate-late (32–36 weeks) preterm.	The number available for MRI was 257 (91 very preterm infants and 166 moderate-late preterm infants); The number available for dMRI was 263 (90 very preterm infants and 173 moderate-late preterm infants);	Prechtl’s assessment of general movements; The Neonatal Intensive Care Unit Network Neurobehavioral Scale (NNNS); The Hammersmith Neonatal Neurological Examination (HNNE)	MRI and dMRI	Suboptimal scores on some assessments were associated with lower fractional anisotropy and/or higher axial, radial, and mean diffusivities in some tracts: NNNS attention and reflexes, and HNNE total score and tone, were associated with the corpus callosum and optic radiation; NNNS quality of movement with the corona radiata; HNNE abnormal signs with several major tracts. Brain structure-function associations generally did not differ between the very and moderate-late preterm groups.
Longitudinal study of neonatal brain tissue volumes in preterm infants and their ability to predict neurodevelopmental outcome (61)	At birth and TEA (MRI are only collected at this stage), 18−24 months, 5 years		84 preterm births at birth and TEA; 74 preterm births at 18−24 months; 56 preterm births at 5 years	At 18−24 months: mental and psychomotor developmental indices (MDI and PDI, respectively) of the Bayley Scales of Infant Development II; At 5 years: French version of the Kaufman Assessment Battery for Children (K-ABC)	MRI acquired at birth and TEA.	From birth to TEA, relative volumes of cortical gray matter (CGM), cerebellum (CB) and cerebrospinal fluid (CSF) with respect to total intracranial volume increased, while relative volumes of UWM and SGM decreased. The fastest growing tissues between birth and TEA were found to be the CB and the CGM. Lower GA at birth was associated with lower growth rates of CGM, CB and total tissue. Among perinatal factors, persistent ductus arteriosus was associated with lower subcortical gray matter (SGM), CB and intracranial (IC) growth rates, while sepsis was associated with lower CSF and intracranial volume growth rates. Model comparisons showed that brain tissue volumes at birth and at TEA contributed to the prediction of motor outcomes at 18−24 months, while volumes at TEA and volume growth rates contributed to the prediction of cognitive scores at 5 years of age. The family socioeconomic status (SES) was not correlated with brain volumes at birth or at TEA, but was strongly associated with the cognitive outcomes at 18−24 months and 5 years of age.

Among the studies mentioned above, there are no study in the age group of 3 to 6 years old, and most studies have a sample size of less than 100 people. From the brain imaging database, brain imaging data of children aged 3−6 is rarely collected, and even few NDDs samples are available. Due to differences in research methods, ages, and research tools, there is no consistent conclusion from the perspective of protective factors and risk factors. Early identification and treatment facilitate the prognosis of DCD and other NDDs. Meanwhile, the systematic evaluation of perinatal factors, DCD detection rate, fMRI, motor development assessment, and other factors is crucial for the healthy development of children with neurodevelopmental disorders under perinatal factors.

Some large cohort study databases are increasingly incorporating motor development data, perinatal and neurodevelopment factors, and brain imaging data from children, such as GUSTO (62) and the FinnBrain Birth Cohort Study (63), which track participants for several years from prenatal and monitoring their brain, cognitive, and behavioral development. Such studies are helpful for understanding the relationship between perinatal factors, motor development, and brain development. However, these cohorts are not publicly available, and it is not clear whether they have assessed motor development and other NDD risk factors, Limiting the possibility of other researchers to use them for more extensive exploration. Some other public child development cohorts did not start to monitor brain development with imaging technology until children were around year 10 years old, including the ABCD study (64) and NCANDA (65), which cannot fully cover the high-risk period (3−6 years) of NDDs. Considering the complexity of human brain development and the high incidence of NDD, it is necessary to conduct larger sample and more targeted cohort studies.

## PeriCBD’s outlook for the field

In view of above, a group of Chinese experts in the fields of psychology, pediatrics, cognitive neuroscience, and neuroimaging jointly launched the project “Multicenter Database on Perinatal Factors in Child Brain-Mind Development” (PeriCBD). We hope to explore the significance of the PeriCBD database in predicting NDDs based on brain imaging data, motor development data, and DCD detection rate. By further verifying the relationship between abnormal development of children’s neural network and various types of NDDs, through the comparison between the experimental group and control group, we hope to establish an early diagnosis model and a multimodal rapid assessment model for different types of NDDs, taking the level of motor development, perinatal adverse factors, and other sensitive indicators as core indications, in order to realize the early rapid screening and diagnosis of children’s NDDs. By the understanding and mastering the brain and cognitive mechanism of NDDs, attempts should be made to intervene for children with NDDs.

Through a series of preparations, the PeriCBD project carried out data collection in 2021 with two county-level hospitals in China. We selected children aged 3−10 years old who had experienced preterm birth (<37 weeks gestation) and low birth weight (<2500 g) as the experimental group of adverse perinatal factors, and recruited ordinary children with the same age and demographic indicators as control. Data of children’s brain magnetic resonance imaging, demographic factors, cognitive ability, motor development, intelligence, detection rate of multiple NDDs disorders, behavioral index, family parenting style, maternal risk factors, maternal mood and others were collected through self-assessment, other assessments, standardized assessments and other methods. Relevant data have been preprocessed and are under further analysis.

In general, with the unity of the definition of DCD, research in this field has steadily increased in the past 10 years, especially in populous and developing countries. This trend is increasingly evident given the importance of motor development in child development. At present, research in this field pays more and more attention to the combination of multidisciplinary, multimodal data such as brain imaging data, perinatal factors, biomarkers, and conventional behavioral data. It is certain that, with the support of mass comprehensive data, the subtype classification and mechanism of DCD can be discussed, and the practical activities such as monitoring, diagnosis, prevention, and intervention of DCD, can be well guided in the near future.

## Author contributions

YW, LK, and XS contributed to the discussion and design of the study. ZD and SH performed the statistical analysis. SY wrote the first draft of the manuscript. LK and XS wrote the manuscript. All authors contributed to the article and approved the submitted version.
